# What predicts large vessel occlusion in mild stroke patients?

**DOI:** 10.1186/s12883-022-03020-6

**Published:** 2023-01-19

**Authors:** Zhengzhao Lu, Yunyun Xiong, Kaixuan Yang, Hongqiu Gu, Chunmiao Duan, Xingquan Zhao, Xia Meng, Yongjun Wang

**Affiliations:** 1 Department of Neurology, Beijing Tiantan Hospital, Capital Medical University, Beijing, China; 2 China National Clinical Research Center for Neurological Diseases, Beijing, China; 3 Chinese Institute for Brain Research, Beijing, China; 4 National Center for Healthcare Quality Management in Neurological Diseases, Beijing, China; 5 Department of Neurology, Beijing Daxing District People’s Hospital, Capital Medical University, Beijing, China; 6 Research Unit of Artificial Intelligence in Cerebrovascular Disease, Chinese Academy of Medical Sciences, 2019RU018, Beijing, China

**Keywords:** Emergency medical services, Thrombectomy, Ischemic stroke, Epidemiology, Clinical decision making

## Abstract

**Background and purpose:**

Mild acute ischemic stroke (AIS) patients with large vessel occlusion (LVO) may benefit from thrombolysis or thrombectomy therapy. However, the predictors for LVO in mild AIS patients have not been extensively explored. We aimed to investigate the predictors for LVO in mild AIS patients.

**Methods:**

We collected the data of consecutive AIS patients with a National Institutes of Health Stroke Scale (NIHSS) score ≤ 5 from The Third China National Stroke Registry - a prospective nationwide registry of AIS or transient ischemic attack (TIA) patients in China from August 2015 to March 2018. Patients were divided into LVO and non-LVO group based on the vascular imaging during the hospitalization. Multivariable regression analyses involving clinical characteristics and NIHSS subitems was performed to detect the predictors for LVO.

**Result:**

A total of 7653 mild AIS patients from The Third China National Stroke Registry were included in this study. Among them, 620 patients (8.1%) had LVO. The level of consciousness (adjusted odds ratio, 1.87; 95% confidence interval, 1.08 to 3.23), visual field (adjusted odds ratio, 2.10; 95% confidence interval, 1.43 to 3.06) and sensory (adjusted odds ratio, 0.75; 95% confidence interval, 0.60 to 0.94) were predictors for mild AIS patients with LVO.

**Conclusions:**

Impaired LOC, visual field and sensory were independently predictors for LVO in mild stroke patients. Further studies are warranted to test these predictors in prehospital setting and in other population.

**Supplementary Information:**

The online version contains supplementary material available at 10.1186/s12883-022-03020-6.

## Introduction

Stroke was the leading cause of disability and mortality at the national level in China [1]. Mild strokes (defined as a National Institutes of Health Stroke Scale [NIHSS] score ≤ 5) [2] accounted for about 47% of all acute ischemic stroke (AIS). Although mild AIS patients were more likely to have a more favorable functional outcome (defined as modified Rankin score [mRS] 0–2) than moderate-to-severe stroke patients, about 30% of mild stroke patients still suffered unfavorable 90-day functional outcome (mRS 3–6) [3].

Large vessel occlusion (LVO) has been proved to be an independent predictor for 6-month mortality and poor functional outcome in AIS patients [4]. The American Stroke Association/American Heart Association (ASA/AHA) guideline recommended endovascular therapy (EVT) as the standard treatment for moderate-to-severe AIS patients with LVO and rapid EVT was able to effectively improve functional outcomes [5]. Mild AIS patients with LVO had higher frequency of early neurological deterioration and a worse functional outcome than non-LVO patients [6]. However, there is no strong evidence of the efficacy of intravenous thrombolysis (IVT) and EVT in this group patients. Currently, the combined treatment of clopidogrel and aspirin for 21 days is the first option of mild non-disabling AIS patients [7], and the expert consensus statement of European Stroke Organization guidelines recommended IVT as the early management [8]. Some retrospective studies showed that EVT reduced the NIHSS score at discharge [9] and improved the rates of independence at 3 months [10] in mild AIS patients with LVO, and the efficacy of EVT is highly dependent on time. Since EVT is only available in some comprehensive stroke centers, these centers are optimal decision for patients with LVO strokes. However, these centers are less and may be hours away. The transfers between two hospitals may delay or prevent the advanced treatment [11]. Therefore, rapid detection of LVO patients were essential.

There are several potential applications for these scales. (1) Patients with suspected LVO who test positive in the field could be directed transported to EVT providing hospitals. (2) The EVT team can be notified earlier when the patient is being transported to an EVT providing hospital. (3) Hospitals without computed tomography angiography (CTA) or magnetic resonance angiography (MRA) capabilities can transport patients with a suspected LVO stroke who have been already received to the EVT providing hospitals. (4) Physicians can identify patients at high risk of early deterioration and optimize the patient pathways [12–14].

NIHSS score was considered to be an independent predictor for LVO [15], and was used in 2/3 studies targeting to derive the predictive scales for LVO [16] in AIS. Consequently, mild AIS patients with LVO are more likely to be neglected and not expeditiously treated by recanalization therapies [17]. Recently, a small sample sized study in mild AIS patients in France compared the clinical symptoms between LVO and non-LVO patients but found no significant predictors for proximal LVO [18]. Thus, we aimed to explore the predictors for LVO in mild AIS patients in a nation-wide prospective registry.

## Method

### Subjects

We included eligible patients from The Third China National Stroke Registry (CNSR-III). The registry was approved by the Central Institutional Review Board in Beijing Tiantan Hospital (IRB approval number: KY2015–001-01), and written informed consents were obtained from all participants. Regarding vulnerable participants, the registry obtained written informed consents from their legally authorized representatives for all vulnerable participants.

The protocol of CNSR-III has been published previously [19]. In brief, CNSR-III was a nationwide prospective registry, which enrolled 15,166 AIS and transient ischemic attack (TIA) patients with detailed information on demography (age and sex), clinical characteristics (medical history, therapy, admission blood pressure, admission NIHSS), imaging and clinical outcomes (stroke recurrence, mRS) in China from August 2015 to March 2018.

In the current study, we included patients who had a mild AIS (defined as total NIHSS score ≤ 5 at admission). The NIHSS score consisted of 11 subitems, which included level of consciousness (LOC), best gaze, visual field, facial palsy, motor arm, motor leg, limb ataxia, sensory, language, dysarthria and neglect. For each subitem, a score of 0 mean normal in this specific function, while a higher score indicated more severe impairment. The scores of each subitem were summed to calculate the NIHSS total score, ranging from 0 to 42. Higher NIHSS score is strongly correlated with more severe stroke. The NIHSS score at admission was assessed by neurology physicians in the emergency department with each subitem score of NIHSS documented. The patients with following criteria were excluded: (1) imaging sequences / quality did not meet the requirement for LVO assessment; (2) the subitems of NIHSS scores were incomplete.

### LVO evaluation

LVO was evaluated by CTA, MRA or digital subtraction angiography (DSA). The imaging core lab performed all the assessment of vascular imaging and LVO. The location of LVO included internal carotid arteries (ICA), A1 segment of anterior cerebral arteries (ACA), M1 segment of middle cerebral arteries (MCA), vertebral arteries (VA), P1 segment of posterior cerebral arteries (PCA) and the basal artery (BA). Among them, ICA, ACA, MCA were parts of the anterior circulation and VA, BA, PCA were parts of the posterior circulation.

### Statistical analysis

The patients were divided into two groups according to whether they had LVO or not. Continuous variables at baseline were shown as the mean ± standard deviation (SD) or as the median (interquartile range, IQR), as appropriate. The difference between two groups was analyzed by using the independent sample t tests or mann-whitney U test. We classified the NIHSS subitems score as 0 and ≥ 1. The categorical variables at baseline and subitems were reported as number (percentages) and were compared by using Fisher exact tests, Chi-square tests or trend tests. Significant variables (*P* value < 0.1) in the univariable analyses were further included in the multivariable logistic regression model. Adjusted odds ratios (ORs) with their 95% confidence intervals (CIs) were calculated. To further investigate the relationship between the motor symptoms and LVO, we combined the NIHSS subitems of motor arm-left and motor arm-right, and the subitems of motor leg-left and motor leg-right. The univariable and multivariable analysis were calculated. The subgroups of anterior and posterior circulation LVO were also analyzed using similar statistical method.

All *P* values presented are two-sided. *P* < 0.05 was considered statistically significant. All analyses were performed using SAS V.9.4 software (SAS Institute, Inc., Cary, NC).

## Result

**Fig. 1:**
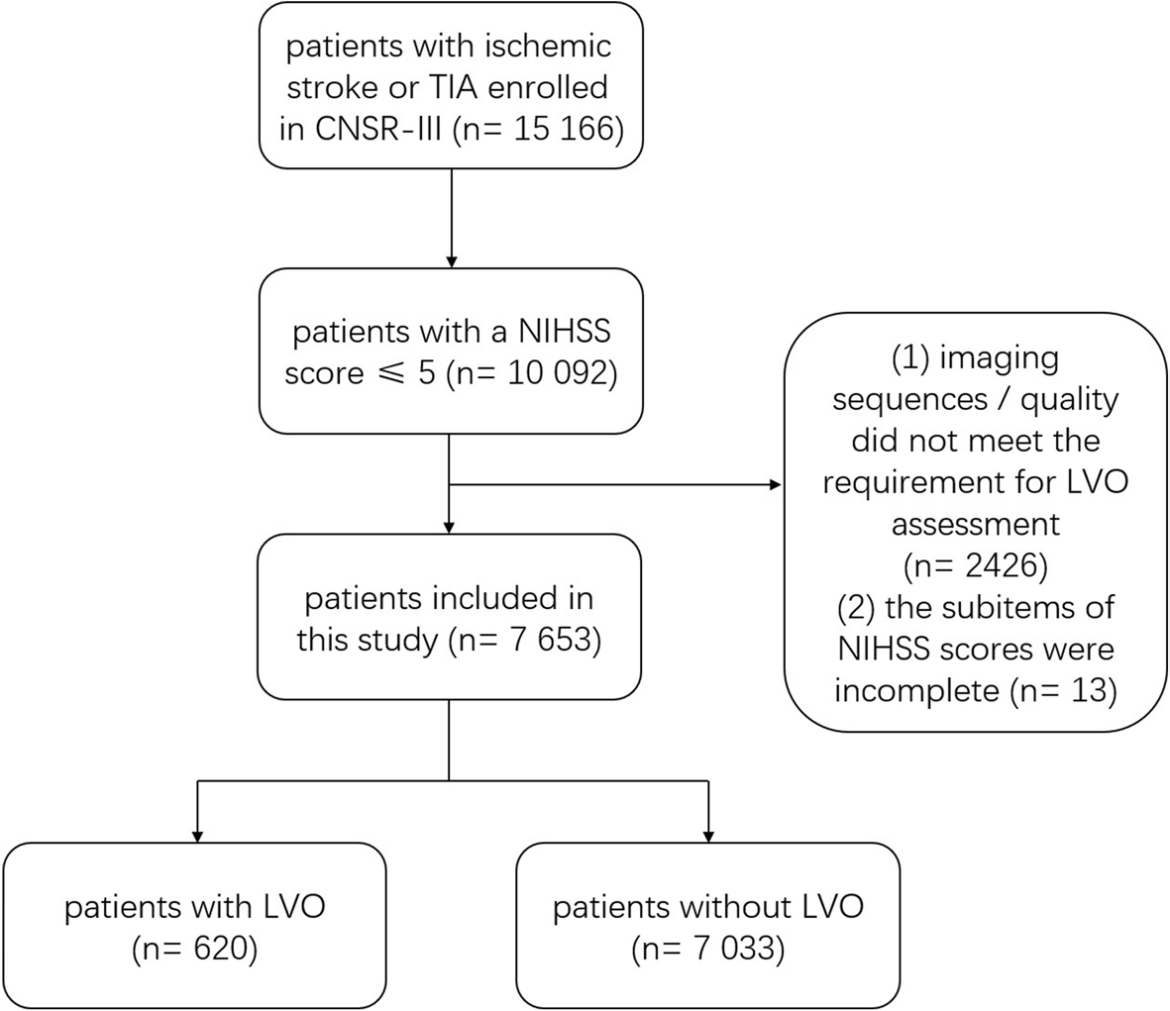
Flowchart of patient recruitment. TIA, transient ischemic attack; CNSR-III, The Third China National Stroke Registry; NIHSS, National Institutes of Health Stroke Scale; LVO, large vessel occlusion

A total of 15,166 patients were enrolled in the CNSR-III study. Among them, 10,092 patients got a mild AIS (NIHSS ≤5). Overall, 2439 patients were excluded according to the exclusion criteria: 2426 patients didn’t have imaging sequences / quality allowing the assessment of LVO, 13 patients had missing NIHSS subitems scores. Finally, 7653 patients were included in this study (Fig. 1). All patients enrolled completed at least one angiography assessment. Among them, 6856 of 7653 patients were assessed by MRA, 767 patients were assessed by CTA, and 30 patients were assessed by DSA.

*LVO* Large vessel occlusion, *SD* Standard deviation, *IQR* Interquartile range, *TIA* Transient ischemic attack, *mRS* Modified Rankin Scale, *NIHSS* National Institutes of Health Stroke Scale, *SBP* Systolic Blood Pressure, *DBP* Diastolic Blood Pressure.

Table 1 summarizes the demographics and clinical characteristics of LVO and non-LVO group. Among all 7653 patients with mild AIS, 620 patients (8.1%) had LVO. The LVO group had more frequent history of prior stroke or TIA (28.2% versus [vs] 22.5%, *p* = 0.001), with a higher admission NIHSS score (3.0 vs 2.0, *p* = 0.017) and a higher admission diastolic blood pressure (DBP) (85.0 vs 86.5, *p* = 0.024) than the non-LVO group. The distribution of LVO locations was shown in Supplementary Table S1.

**Table 1:** Demographics and clinical characteristics of mild AIS patients with or without LVO

Variables	Total (***N*** = 7653)	LVO group (***N*** = 620 [8.1%])	non-LVO group (***N*** = 7033 [91.9%])	***P*** Value
**Demographic**				
**Age, Mean ± SD, y**	**62.0 ± 11.2**	**62.8 ± 11.7**	**62.0 ± 11.1**	**0.096**
**Male, n (%)**	**5349 (69.9)**	**436 (70.3)**	**4913 (69.9)**	**0.808**
**Medical history**				
**Diabetes mellitus, n (%)**	**1784 (23.3)**	**147 (23.7)**	**1637 (23.3)**	**0.807**
**Hypertension, n (%)**	**4830 (63.1)**	**387 (62.4)**	**4443 (63.2)**	**0.71**
**Dyslipidemia, n (%)**	**613 (8.0)**	**45 (7.3)**	**568 (8.1)**	**0.472**
**Prior Stroke or TIA, n (%)**	**1758 (23.0)**	**175 (28.2)**	**1583 (22.5)**	**0.001**
**Prior Stroke, n (%)**	**1624 (21.2)**	**157 (25.3)**	**1467 (20.9)**	**0.009**
**Myocardial infarction, n (%)**	**112 (1.5)**	**5 (0.8)**	**107 (1.5)**	**0.155**
**Atrial fibrillation, n (%)**	**200 (2.6)**	**17 (2.7)**	**183 (2.6)**	**0.834**
**Smoking, n (%)**	**3484 (45.5)**	**278 (44.8)**	**3206 (45.6)**	**0.721**
**Drinking, n (%)**	**3519 (46.0)**	**282 (45.5)**	**3237 (46.0)**	**0.795**
**Pre mRS, n (%)**				**0.079**
**0–2**	**7452 (97.4)**	**597 (96.3)**	**6855 (97.5)**	
**3–5**	**201 (2.6)**	**23 (3.7)**	**178 (2.5)**	
**Hospital admission**				
**Admission NIHSS**	**2.0 (1.0–4.0)**	**3.0 (1.0–4.0)**	**2.0 (1.0–4.0)**	**0.017**
**Admission SBP, median (IQR), mmHg**	**148.5 (135.0–163.0)**	**147.0 (133.3–163.0)**	**149.0 (135.0–163.5)**	**0.282**
**Admission DBP, median (IQR), mmHg**	**86.5 (79.5–95.5)**	**85.0 (79.0–93.5)**	**86.5 (79.5–95.5)**	**0.024**
**Endovascular Therapy, n (%)**	**16 (0.2)**	**3 (0.5)**	**13 (0.2)**	**0.118**
**Intravenous Thrombolysis, n (%)**	**19 (0.2)**	**5 (0.8)**	**14 (0.2)**	**0.004**
**Median time from onset to NIHSS assessment, median (IQR), hours**	**17.0 (5.0–43.5)**	**20.0 (5.0–48.6)**	**17.0 (5.0–41.6)**	**0.053**
**Median time from onset to imaging, median (IQR), hours**	**48.0 (24.0–96.0)**	**72.0 (24.0–96.0)**	**48.0 (24.0–96.0)**	**0.031**

Table 2 shows the univariate analysis and multivariate analysis of each NIHSS subitems. In the univariate analysis, patients with LVO were more common in symptoms of LOC (crude OR, 2.04; 95% CI, 1.19 to 3.50, *p* = 0.008), LOC questions (crude OR, 1.56; 95% CI, 0.93 to 2.60, *p* = 0.088), LOC commands (crude OR, 1.91; 95% CI, 1.00 to 3.63, *p* = 0.046), visual field (crude OR, 2.21; 95% CI, 1.52 to 3.22, *p* < .0001), left arm motor (crude OR, 1.36; 95% CI, 1.11 to 1.65, *p* = 0.002), right arm motor (crude OR, 0.74; 95% CI, 0.59 to 0.94, *p* = 0.013), left leg motor (crude OR, 1.29; 95% CI, 1.06 to 1.56, *p* = 0.010), limb ataxia (crude OR, 1.21; 95% CI, 0.97 to 1.52, *p* = 0.095) and lower subitem scores in sensory (crude OR, 0.73; 95% CI, 0.58 to 0.91, *p* = 0.005). Age, sex and other predictive factors with *P* value < 0.1 were further included in the multivariate model. The multivariate model yielded the LOC (adjusted OR, 1.87; 95% CI, 1.08 to 3.23, *p* = 0.025), visual field (adjusted OR, 2.09; 95% CI, 1.43 to 3.06, *p* < .0.001) and sensory (adjusted OR, 0.75; 95% CI, 0.60 to 0.94, *p* = 0.014) as independent predictors for LVO. The distribution of the NIHSS subitem scores was shown in Supplementary Table S2.

**Table 2:** The univariate and multivariate analyses of each NIHSS subitems between the LVO and non-LVO group in mild AIS patients

NIHSS subitem	Crude OR^a^	***P*** Value (univariate model)	Adjusted OR^a,b^	***P*** Value (multivariate model)
**Level of consciousness**	**2.04 (1.19–3.50)**	**0.008**	**1.87 (1.08–3.23)**	**0.025**
**Consciousness Questions**	**1.56 (0.93–2.60)**	**0.088**	**1.27 (0.72–2.23)**	**0.412**
**Consciousness Commands**	**1.91 (1.00–3.63)**	**0.046**	**1.50 (0.74–3.04)**	**0.258**
**Best Gaze**	**1.26 (0.63–2.53)**	**0.507**		
**Visual Field**	**2.21 (1.52–3.22)**	**<.0001**	**2.09 (1.43–3.06)**	**<.0.001**
**Facial Palsy**	**1.09 (0.92–1.29)**	**0.318**		
**Motor Arm-left**	**1.36 (1.11–1.65)**	**0.002**	**1.29 (0.98–1.71)**	**0.067**
**Motor Arm-right**	**0.74 (0.59–0.94)**	**0.013**	**0.81 (0.63–1.03)**	**0.085**
**Motor Leg-left**	**1.29 (1.06–1.56)**	**0.010**	**1.04 (0.80–1.36)**	**0.783**
**Motor Leg-right**	**0.88 (0.71–1.08)**	**0.219**		
**Limb Ataxia**	**1.21 (0.97–1.52)**	**0.095**	**1.24 (0.98–1.56)**	**0.068**
**Sensory**	**0.73 (0.58–0.91)**	**0.005**	**0.75 (0.60–0.94)**	**0.014**
**Language**	**1.14 (0.94–1.40)**	**0.185**		
**Dysarthria**	**1.05 (0.87–1.25)**	**0.625**		
**Neglect**	**0.97 (0.30–3.17)**	**0.963**		

*NIHSS* National Institutes of Health Stroke Scale, *LVO* Large vessel occlusion, *OR* Odds ratio.

^a^ORs were calculated based on whether the symptom occurred ^b^adjusted for age, sex, prior stroke or TIA, diastolic blood pressure

Sensitivity tests results in the anterior and posterior circulation LVO separately were shown in Tables 3 and 4. In the multivariable logistic regression model of anterior circulation LVO, we found the anterior circulation LVO was correlated positively with facial palsy (adjusted OR, 1.57; 95% CI, 1.26 to 1.97, *p* < .001), left arm motor (adjusted OR, 1.63; 95% CI, 1.15 to 2.31, *p* = 0.006), aphasia (adjusted OR, 1.55; 95% CI, 1.22 to 1.99, *p* < .001), and inversely with age (adjusted OR, 0.99; 95% CI, 0.98 to 0.99, *p* = 0.023), admission DBP (adjusted OR, 0.99; 95% CI, 0.98 to 0.99, *p* = 0.001) and sensory (adjusted OR, 0.51; 95% CI, 0.36 to 0.72, *p* < .001). By contrast, in the posterior circulation LVO group, LVO was significantly positively correlated with age (adjusted OR, 1.02; 95% CI, 1.01 to 1.04, *p* < .001), LOC (adjusted OR, 2.52; 95% CI, 1.25 to 5.10, *p* = 0.010), visual field (adjusted OR, 3.12; 95% CI, 1.98 to 4.93, *p* < .001), limb ataxia (adjusted OR, 1.58; 95% CI, 1.16 to 2.14, *p* = 0.003) and inversely with facial palsy (adjusted OR, 0.65; 95% CI, 0.49 to 0.85, *p* = 0.002) and aphasia (adjusted OR, 0.62; 95% CI, 0.43 to 0.89, *p* = 0.010). Table S3, Table S4 and Table S5 in the supplementary material showed the analysis combined the left and right motor symptoms, and the results remained the same.

**Table 3:** The univariate and multivariate analyses of each NIHSS subitems between the anterior LVO and anterior non-LVO group in mild AIS patients

NIHSS subitem	Crude OR^a^	***P*** Value (univariate model)	Adjusted OR^a,b^	***P*** Value (multivariate model)
**Level of consciousness**	**1.73 (0.84–3.59)**	**0.136**		
**Consciousness Questions**	**1.79 (0.96–3.34)**	**0.065**	**1.47 (0.73–2.94)**	**0.277**
**Consciousness Commands**	**2.12 (0.97–4.65)**	**0.054**	**1.77 (0.74–4.20)**	**0.198**
**Best Gaze**	**1.24 (0.50–3.07)**	**0.644**		
**Visual Field**	**1.37 (0.77–2.43)**	**0.277**		
**Facial Palsy**	**1.67 (1.34–2.07)**	**<.001**	**1.57 (1.26–1.97)**	**<.001**
**Motor Arm-left**	**1.84 (1.44–2.34)**	**<.001**	**1.63 (1.15–2.31)**	**0.006**
**Motor Arm-right**	**077 (0.57–1.05)**	**0.103**		
**Motor Leg-left**	**1.61 (1.27–2.04)**	**<.0001**	**1.20 (0.85–1.70)**	**0.291**
**Motor Leg-right**	**0.86 (0.65–1.13)**	**0.272**		
**Limb Ataxia**	**0.89 (0.64–1.24)**	**0.488**		
**Sensory**	**0.49 (0.35–0.68)**	**<.001**	**0.51 (0.36–0.72)**	**<.001**
**Language**	**1.68 (1.33–2.14)**	**<.001**	**1.55 (1.22–1.99)**	**<.001**
**Dysarthria**	**1.29 (1.03–1.62)**	**0.029**	**1.13 (0.89–1.43)**	**0.329**
**Neglect**	**1.81 (0.55–5.90)**	**0.321**		

*NIHSS* National Institutes of Health Stroke Scale, *LVO* Large vessel occlusion, *OR* Odds ratio.

^a^ORs were calculated based on whether the symptom occurred

^b^adjusted for age, sex, prior stroke or TIA, diastolic blood pressure

**Table 4:** The univariate and multivariate analyses of each NIHSS subitems between the posterior LVO and posterior non-LVO group in mild AIS patients

NIHSS subitem	Crude OR^a^	***P*** Value (univariate model)	Adjusted OR^a,b^	***P*** Value (multivariate model)
**Level of consciousness**	**2.48 (1.24–4.97)**	**0.008**	**2.52 (1.25–5.10)**	**0.010**
**Consciousness Questions**	**1.16 (0.51–2.65)**	**0.723**		
**Consciousness Commands**	**1.44 (0.52–3.98)**	**0.475**		
**Best Gaze**	**1.23 (0.45–3.36)**	**0.695**		
**Visual Field**	**3.35 (2.14–5.26)**	**<.001**	**3.12 (1.98–4.93)**	**<.001**
**Facial Palsy**	**0.58 (0.45–0.79)**	**<.001**	**0.65 (0.49–0.85)**	**0.002**
**Motor Arm-left**	**0.86 (0.62–1.19)**	**0.358**		
**Motor Arm-right**	**0.69 (0.49–0.99)**	**0.042**	**0.74 (0.52–1.05)**	**0.094**
**Motor Leg-left**	**0.93 (0.69–1.25)**	**0.627**		
**Motor Leg-right**	**0.90 (0.66–1.22)**	**0.491**		
**Limb Ataxia**	**1.61 (1.20–2.18)**	**0.002**	**1.58 (1.16–2.14)**	**0.003**
**Sensory**	**1.13 (0.85–1.51)**	**0.399**		
**Language**	**0.57 (0.40–0.81)**	**0.002**	**0.62 (0.43–0.89)**	**0.010**
**Dysarthria**	**0.78 (0.59–1.03)**	**0.075**	**0.91 (0.68–1.21)**	**0.507**
**Neglect**	-**	**0.228**		

*NIHSS* National Institutes of Health Stroke Scale, *LVO* Large vessel occlusion, *OR* Odds ratio.

^a^ORs were calculated based on whether the symptom occurred

^b^adjusted for age, sex, prior stroke or TIA, diastolic blood pressure

^c^OR value was incalculable because the incidence of neglect in LVO group was zero

## Discussion

Our study found that approximate 8.1% of mild AIS patients had an occlusion of anterior and posterior large vessels. Patients with a history of prior TIA or stroke, higher NIHSS score and higher admission DBP were more likely to get LVO. In the subitems of NIHSS score, we found that LOC, visual field and sensory were independent predictors for LVO in mild AIS patients.

A non-contrast computed tomography was recommended for mild AIS patients in the most current AHA/ASA guideline of stroke [13]. Therefore, the LVO patients with mild symptoms may miss the angiography examination and delay the EVT therapy. Previous studies showed that prehospital prediction scales of LVO could shorten the onset-to-puncture time at EVT providing hospitals [4]. Our study investigated the possibility to build a prediction LVO model in mild AIS patients, and discovered several symptoms may be helpful to identify LVO in the clinical practice.

Our study was partly consistent with previous studies in the population of all AIS patients [20–22]. In previous studies [20–22], the LOC, aphasia, gaze palsy, neglect and hemianopia were all considered as cortical symptoms which were highly associated with LVO in the stoke population. Recently, a retrospective study from France indicated that mild AIS patients with LVO were more prone to have higher score on consciousness and aphasia, although in the multivariable analysis these differences were not significant [18]. Therefore, most of prehospital prediction scales of LVO (e.g., 3I-SS [22], FAST-ED [21], RACE [23]) included at least two of these symptoms. Our study found the LOC and hemianopia as predictors with similar risk in mild AIS patients. The differences were not statistically significant in other symptoms probably because of two reasons. Firstly, the incidence of these symptoms in this study was lower than other studies. Only 90 (1.2%) of patients had partial gaze palsy or forced eye deviation, and 38 (0.5%) of patients had neglect symptom. Mild symptoms of gaze palsy and neglect were defined as non-disabling symptoms [24]. Therefore, the frequency of these patients to go to hospitals was lower than other symptoms such as motor problem or hemianopsia. Moderate-to-severe patients often have complex clinical symptoms, resulting in a higher detection rate in these non-disabling symptoms. Secondly, the presentation of ischemic stroke with isolated aphasia symptom is more common in mild AIS patients and often reflects the occlusions of distal vessels [25], which might explain the high incidence of aphasia in non-LVO group.

We found a significantly converse correlation between sensory and LVO in mild AIS patients. Conversely, no relationship between them had been indicated in previous studies [20–22]. This was most likely due to the high incidence of pure hemisensory loss syndrome, which accounted for about 3.5% of mild AIS patients and had been identified to be a strong positive predictor for lacunar infarction [26]. However, none of current prediction scales took sensory symptom under consideration, which might lead to inaccuracies in the prediction for LVO in mild AIS patients.

Compared with these cortical symptoms, motor problems also happen in lacunar stroke and might not be a reliable predictor for LVO, whereas most of scales included motor symptoms as an essential item and gave it a high weight in assignment [27]. One possible reason is that severe motor symptom is associated with large lesion in cortex [28], which is a strong predictor for LVO. Our study only found motor symptoms were correlated with anterior circulation, but not overall LVO. Previous studies showed the less portion of motor problem in posterior circulation infarctions than in anterior circulation, which hampered the analysis of correlation between posterior circulation LVO and motor problem.

Many scales based on FAST (Face Arm Speech Test) included facial palsy as one of the predictors for LVO. Our studies partially agreed with this view. We discovered that facial palsy was positively correlated with anterior LVO, but conversely correlated with posterior LVO. This might explain the worse predictive capability of FAST scale for the severity of stroke in posterior circulation [29].

In this study, there were differences in the prediction symptoms for LVO between the anterior and posterior circulation. These differences found in LVO patients were consistent with previous studies in other population [14, 15]. In the analysis of the anterior circulation, the dysfunction of sensory, aphasia and motor symptoms were related to the infarction in the cortex, which were less common found in the subcortical infarction caused by perforator artery occlusion. The higher frequency of visual fields, limb ataxia and LOC in posterior LVO patients demonstrated a large infarct in the occipital lobe, cerebellum and brainstem. Some study had demonstrated that the subitems of NIHSS may not be suitable for the posterior circulation ischemic stroke [16]. Symptoms like vertigo and imbalance have a stronger specificity in posterior circulation infarction [17], which may need further research.

Our study had several limitations. Firstly, our study excluded thrombolysis patients who didn’t have angiography evaluation before thrombolysis, and without LVO on angiography after thrombolysis, because we couldn’t evaluate whether they had an occlusion before thrombolysis or not. All of these patients received thrombolysis based on physicians’ own experience, judgment, and technical abilities. Consequently, these patients might have more severe symptoms than non-thrombolysis patients. The exclusion of these patients might underestimate the actual prevalence of LVO in mild AIS patients. This population accounted for less than 10% of overall, and further interpretation of our results needs to be cautious. Secondly, considering that the median time from onset to assessment in our study was 17 hours, the results did not necessarily apply to hyperacute stroke patients. Thirdly, due to the ceiling effect of NIHSS in mild AIS, some NIHSS subitems did not have scores. However, our study is the largest prospective study of mild AIS patients with LVO. Therefore, our study shed light on the predictors for LVO, further studies in other ethnics are needed to confirm or refute our findings.

## Conclusion

Impaired LOC, visual field and sensory were independently predictors for LVO in mild stroke patients. Further studies are warranted to test these predictors in prehospital setting and in other population.

## Supplementary Information

**Additional file 1: Table S1.** The distribution of LVO locations in mild AIS patients.

**Additional file 2: Table S2.** The distribution of the NIHSS subitem scores in mild AIS patients.

**Additional file 3: Table S3.** The univariate and multivariate analyses of each NIHSS subitems with combined left and right motor symptoms between the LVO and non-LVO group in mild stroke patients.

**Additional file 4: Table S4.** The univariate and multivariate analyses of each NIHSS subitems with combined left and right motor symptoms between the anterior LVO and anterior non-LVO group in mild stroke patients.

**Additional file 5: Table S5.** The univariate and multivariate analyses of each NIHSS subitems with combined left and right motor symptoms between the posterior LVO and posterior non-LVO group in mild stroke patients.

## Data Availability

The data that support the findings of this study are available from the corresponding author but restrictions apply to the availability of these data, which were used under license for the current study, and so are not publicly available. Data are however available from the authors upon reasonable request and with permission of the corresponding author.
