# Supplementary material for: What predicts large vessel occlusion in mild stroke patients?
Source: BMC Neurol. 2023 Jan 19;23:29. doi: 10.1186/s12883-022-03020-6 (PMC9850683; doi:10.1186/s12883-022-03020-6)
Supplement: Supplementary file 1 — Additional file 1: Table S1. The distribution of LVO locations in mild AIS patients. [file 12883_2022_3020_MOESM1_ESM.docx]

| **Table S1: the distribution of LVO locations in mild AIS patients** | |
| --- | --- |
| **the locations of LVO** | **Number (%)** |
| **Internal carotid artery (ICA)** | **101 (16.3)** |
| **anterior cerebral artery (ACA)** | **37 (6.0)** |
| **middle cerebral artery (MCA)** | **201 (32.4)** |
| **posterior cerebral artery (PCA)** | **90 (14.5)** |
| **vertebral artery (VA)** | **120 (19.4)** |
| **basilar artery (BA)** | **62 (10.0)** |
| **ICA+VA** | **1 (0.2)** |
| **ICA+BA** | **1 (0.2)** |
| **MCA+BA** | **2 (0.3)** |
| **MCA+PCA** | **3 (0.5)** |
| **ACA+VA** | **1 (0.2)** |
| **ACA+PCA** | **1 (0.2)** |
| **AIS,acute ischemic stroke;LVO, large vessel occlusion;** | |
