# Supplementary material for: What predicts large vessel occlusion in mild stroke patients?
Source: BMC Neurol. 2023 Jan 19;23:29. doi: 10.1186/s12883-022-03020-6 (PMC9850683; doi:10.1186/s12883-022-03020-6)
Supplement: Supplementary file 2 — Additional file 2: Table S2. The distribution of the NIHSS subitem scores in mild AIS patients. [file 12883_2022_3020_MOESM2_ESM.docx]

| **Table S2: the distribution of the NIHSS subitem scores in mild AIS patients** | | | |
| --- | --- | --- | --- |
| **Variables** | **Total (N=7653 [100%])** | **LVO (N=620 [8.1%])** | **Not LVO (N=7033 [91.9%])** |
| **Level of consciousness** |  |  |  |
| 0 | **7547 (98.6)** | **604 (97.4)** | **6943 (98.7)** |
| 1 | **96 (1.3)** | **16 (2.6)** | **80 (1.1)** |
| 2 | **6 (0.1)** |  | **6 (0.1)** |
| 3 | **4 (0.1)** |  | **4 (0.1)** |
| **Consciousness Questions** |  |  |  |
| 0 | **7511 (98.1)** | **603 (97.3)** | **6908 (98.2)** |
| 1 | **106 (1.4)** | **13 (2.1)** | **93 (1.3)** |
| 2 | **36 (0.5)** | **4 (0.6)** | **32 (0.5)** |
| **Consciousness Commands** |  |  |  |
| 0 | **7576 (99.0)** | **609 (98.2)** | **6967 (99.1)** |
| 1 | **70 (0.9)** | **9 (1.5)** | **61 (0.9)** |
| 2 | **7 (0.1)** | **2 (0.3)** | **5 (0.1)** |
| **Best Gaze** |  |  |  |
| 0 | **7563 (98.8)** | **611 (98.5)** | **6952 (98.8)** |
| 1 | **82 (1.1)** | **8 (1.3)** | **74 (1.1)** |
| 2 | **8 (0.1)** | **1 (0.2)** | **7 (0.1)** |
| **Visual Field** |  |  |  |
| 0 | **7439 (97.2)** | **586 (94.5)** | **6853 (97.4)** |
| 1 | **176 (2.3)** | **27 (4.4)** | **149 (2.1)** |
| 2 | **26 (0.3)** | **7 (1.1)** | **19 (0.3)** |
| 3 | **12 (0.2)** |  | **12 (0.2)** |
| **Facial Palsy** |  |  |  |
| 0 | **4551 (59.5)** | **357 (57.6)** | **4194 (59.6)** |
| 1 | **2588 (33.8)** | **218 (35.2)** | **2370 (33.7)** |
| 2 | **500 (6.5)** | **44 (7.1)** | **456 (6.5)** |
| 3 | **14 (0.2)** | **1 (0.2)** | **13 (0.2)** |
| **Motor Arm** |  |  |  |
| 0 | **4999 (65.3)** | **400 (64.5)** | **4599 (65.4)** |
| 1 | **2282 (29.8)** | **184 (29.7)** | **2098 (29.8)** |
| 2 | **304 (4.0)** | **29 (4.7)** | **275 (3.9)** |
| 3 | **46 (0.6)** | **5 (0.8)** | **41 (0.6)** |
| 4 | **5 (0.1)** |  | **5 (0.1)** |
| 9 | **17 (0.2)** | **2 (0.3)** | **15 (0.2)** |
| **Motor Leg** |  |  |  |
| 0 | **4650 (60.8)** | **363 (58.5)** | **4287 (61.0)** |
| 1 | **2469 (32.3)** | **216 (34.8)** | **2253 (32.0)** |
| 2 | **443 (5.8)** | **37 (6.0)** | **406 (5.8)** |
| 3 | **54 (0.7)** | **3 (0.5)** | **51 (0.7)** |
| 4 | **10 (0.1)** | **1 (0.2)** | **9 (0.1)** |
| 9 | **27 (0.4)** |  | **27 (0.4)** |
| **Limb Ataxia** |  |  |  |
| 0 | **6612 (86.4)** | **522 (84.2)** | **6090 (86.6)** |
| 1 | **754 (9.9)** | **74 (11.9)** | **680 (9.7)** |
| 2 | **287 (3.8)** | **24 (3.9)** | **263 (3.7)** |
| **Sensory** |  |  |  |
| 0 | **6142 (80.3)** | **524 (84.5)** | **5618 (79.9)** |
| 1 | **1493 (19.5)** | **96 (15.5)** | **1397 (19.9)** |
| 2 | **18 (0.2)** |  | **18 (0.3)** |
| **Language** |  |  |  |
| 0 | **6142 (80.3)** | **485 (78.2)** | **5657 (80.4)** |
| 1 | **1414 (18.5)** | **122 (19.7)** | **1292 (18.4)** |
| 2 | **87 (1.1)** | **12 (1.9)** | **75 (1.1)** |
| 3 | **10 (0.1)** | **1 (0.2)** | **9 (0.1)** |
| **Dysarthria** |  |  |  |
| 0 | **5496 (71.8)** | **440 (71.0)** | **5056 (71.9)** |
| 1 | **2061 (26.9)** | **172 (27.7)** | **1889 (26.9)** |
| 2 | **76 (1.0)** | **7 (1.1)** | **69 (1.0)** |
| 9 | **20 (0.3)** | **1 (0.2)** | **19 (0.3)** |
| **Neglect** |  |  |  |
| 0 | **7615 (99.5)** | **617 (99.5)** | **6998 (99.5)** |
| 1 | **35 (0.5)** | **2 (0.3)** | **33 (0.5)** |
| 2 | **3 (0.0)** | **1 (0.2)** | **2 (0.0)** |
| **NIHSS, National Institutes of Health Stroke Scale; LVO, large vessel occlusion;** | | | |
