# Supplementary material for: What predicts large vessel occlusion in mild stroke patients?
Source: BMC Neurol. 2023 Jan 19;23:29. doi: 10.1186/s12883-022-03020-6 (PMC9850683; doi:10.1186/s12883-022-03020-6)
Supplement: Supplementary file 3 — Additional file 3: Table S3. The univariate and multivariate analyses of each NIHSS subitems with combined left and right motor symptoms between the LVO and non-LVO group in mild stroke patients. [file 12883_2022_3020_MOESM3_ESM.docx]

| **Table S3: the univariate and multivariate analyses of each NIHSS subitems with combined left and right motor symptoms between the LVO and non-LVO group in mild stroke patients** | | | | |
| --- | --- | --- | --- | --- |
| **NIHSS subitem** | **Crude OR** | **P Value (univariate model)** | **Adjusted OR*** | **P Value (multivariate model)** |
| **Level of consciousness** | **2.04 (1.19-3.50)** | **0.008** | **1.88 (1.09-3.25)** | **0.023** |
| **Consciousness Questions** | **1.56 (0.93-2.60)** | **0.088** | **1.23 (0.70-2.15)** | **0.475** |
| **Consciousness Commands** | **1.91 (1.00-3.63)** | **0.046** | **1.52 (0.75-3.07)** | **0.243** |
| **Best Gaze** | **1.26 (0.63-2.53)** | **0.507** |  |  |
| **Visual Field** | **2.21 (1.52-3.22)** | **<.0001** | **2.09 (1.43-3.06)** | **<.0001** |
| **Facial Palsy** | **1.09 (0.92-1.29)** | **0.318** |  |  |
| **Motor Arm** | **1.04(0.88-1.23)** | **0.659** |  |  |
| **Motor Leg** | **1.11(0.94-1.31)** | **0.240** |  |  |
| **Limb Ataxia** | **1.21 (0.97-1.52)** | **0.095** | **1.25 (1.00-1.57)** | **0.057** |
| **Sensory** | **0.73 (0.58-0.91)** | **0.005** | **0.75 (0.60- 0.94)** | **0.013** |
| **Language** | **1.14 (0.94-1.40)** | **0.185** |  |  |
| **Dysarthria** | **1.05 (0.87-1.25)** | **0.625** |  |  |
| **Neglect** | **0.97 (0.30-3.17)** | **0.963** |  |  |
| **NIHSS, National Institutes of Health Stroke Scale; LVO, large vessel occlusion; OR, odds ratio * adjusted for age, sex, prior stroke or TIA, diastolic blood pressure** | | | | |
