# Supplementary material for: What predicts large vessel occlusion in mild stroke patients?
Source: BMC Neurol. 2023 Jan 19;23:29. doi: 10.1186/s12883-022-03020-6 (PMC9850683; doi:10.1186/s12883-022-03020-6)
Supplement: Supplementary file 4 — Additional file 4: Table S4. The univariate and multivariate analyses of each NIHSS subitems with combined left and right motor symptoms between the anterior LVO and anterior non-LVO group in mild stroke patients. [file 12883_2022_3020_MOESM4_ESM.docx]

| **Table S4: the univariate and multivariate analyses of each NIHSS subitems with combined left and right motor symptoms between the anterior LVO and anterior non-LVO group in mild stroke patients** | | | | |
| --- | --- | --- | --- | --- |
| **NIHSS subitem** | **Crude OR** | **P Value (univariate model)** | **Adjusted OR*** | **P Value (multivariate model)** |
| **Level of consciousness** | **1.73 (0.84-3.59)** | **0.136** |  |  |
| **Consciousness Questions** | **1.79 (0.96-3.34)** | **0.065** | **1.44 (0.72-2.89)** | **0.299** |
| **Consciousness Commands** | **2.12 (0.97-4.65)** | **0.054** | **1.81 (0.76-4.30)** | **0.178** |
| **Best Gaze** | **1.24 (0.50-3.07)** | **0.644** |  |  |
| **Visual Field** | **1.37 (0.77-2.43)** | **0.277** |  |  |
| **Facial Palsy** | **1.67 (1.34-2.07)** | **<.001** | **1.59 (1.27-2.00)** | **<.001** |
| **Motor Arm** | **1.35 (1.08-1.68)** | **0.007** | **1.17 (0.88-1.55)** | **0.290** |
| **Motor Leg** | **1.34 (1.08-1.66)** | **0.009** | **1.25 (0.94-1.66)** | **0.122** |
| **Limb Ataxia** | **0.89 (0.64-1.24)** | **0.488** |  |  |
| **Sensory** | **0.49 (0.35-0.68)** | **<.001** | **0.50 (0.36-0.71)** | **<.001** |
| **Language** | **1.68 (1.33-2.14)** | **<.001** | **1.50 (1.18-1.92)** | **0.001** |
| **Dysarthria** | **1.29 (1.03-1.62)** | **0.029** | **1.11 (0.87-1.40)** | **0.410** |
| **Neglect** | **1.81 (0.55-5.90)** | **0.321** |  |  |
| **NIHSS, National Institutes of Health Stroke Scale; LVO, large vessel occlusion; OR, odds ratio * adjusted for age, sex, prior stroke or TIA, diastolic blood pressure** | | | | |
