# Supplementary material for: What predicts large vessel occlusion in mild stroke patients?
Source: BMC Neurol. 2023 Jan 19;23:29. doi: 10.1186/s12883-022-03020-6 (PMC9850683; doi:10.1186/s12883-022-03020-6)
Supplement: Supplementary file 5 — Additional file 5: Table S5. The univariate and multivariate analyses of each NIHSS subitems with combined left and right motor symptoms between the posterior LVO and posterior non-LVO group in mild stroke patients. [file 12883_2022_3020_MOESM5_ESM.docx]

| **Table S5: the univariate and multivariate analyses of each NIHSS subitems with combined left and right motor symptoms between the posterior LVO and posterior non-LVO group in mild stroke patients** | | | | |
| --- | --- | --- | --- | --- |
| **NIHSS subitem** | **Crude OR** | **P Value (univariate model)** | **Adjusted OR*** | **P Value (multivariate model)** |
| **Level of consciousness** | **2.48 (1.24-4.97)** | **0.008** | **2.54 (1.25-5.12)** | **0.010** |
| **Consciousness Questions** | **1.16 (0.51-2.65)** | **0.723** |  |  |
| **Consciousness Commands** | **1.44 (0.52-3.98)** | **0.475** |  |  |
| **Best Gaze** | **1.23 (0.45-3.36)** | **0.695** |  |  |
| **Visual Field** | **3.35 (2.14-5.26)** | **<.001** | **3.08 (1.95-4.87)** | **<.001** |
| **Facial Palsy** | **0.58 (0.45-0.79)** | **<.001** | **0.66 (0.50-0.87)** | **0.003** |
| **Motor Arm** | **0.73(0.56-0.95)** | **0.019** | **0.76 (0.58-1.00)** | **0.049** |
| **Motor Leg** | **0.86(0.67-1.11)** | **0.249** |  |  |
| **Limb Ataxia** | **1.61 (1.20-2.18)** | **0.002** | **1.58 (1.17-2.14)** | **0.003** |
| **Sensory** | **1.13 (0.85-1.51)** | **0.399** |  |  |
| **Language** | **0.57 (0.40-0.81)** | **0.002** | **0.61 (0.43-0.88)** | **0.008** |
| **Dysarthria** | **0.78 (0.59-1.03)** | **0.075** | **0.90 (0.67-1.20)** | **0.463** |
| **Neglect** | **-**** | **0.228** |  |  |
| **NIHSS, National Institutes of Health Stroke Scale; LVO, large vessel occlusion; OR, odds ratio * adjusted for age, sex, prior stroke or TIA, diastolic blood pressure** | | | | |
